# Roles of IL-1 in Cancer: From Tumor Progression to Resistance to Targeted Therapies

**DOI:** 10.3390/ijms21176009

**Published:** 2020-08-20

**Authors:** Valerio Gelfo, Donatella Romaniello, Martina Mazzeschi, Michela Sgarzi, Giada Grilli, Alessandra Morselli, Beatrice Manzan, Karim Rihawi, Mattia Lauriola

**Affiliations:** 1 Department of Experimental, Diagnostic and Specialty Medicine (DIMES), University of Bologna, 40138 Bologna, Italy; valerio.gelfo2@unibo.it (V.G.); donatella.romaniello@unibo.it (D.R.); martina.mazzeschi2@unibo.it (M.M.); michela.sgarzi2@unibo.it (M.S.); giada.grilli2@unibo.it (G.G.); alessandra.morselli2@studio.unibo.it (A.M.); beatrice.manzan@studio.unibo.it (B.M.); 2 Centre for Applied Biomedical Research (CRBA), Bologna University Hospital Authority St. Orsola-Malpighi Polyclinic, 40138 Bologna, Italy; 3 Department of Oncology, Policlinico S. Orsola-Malpighi, University of Bologna, 40138 Bologna, Italy; karim.rihawi@gmail.com

**Keywords:** IL-1, cancer, resistance

## Abstract

IL-1 belongs to a family of 11 members and is one of the seven receptor-agonists with pro-inflammatory activity. Beyond its biological role as a regulator of the innate immune response, IL-1 is involved in stress and chronic inflammation, therefore it is responsible for several pathological conditions. In particular, IL-1 is known to exert a critical function in malignancies, influencing the tumor microenvironment and promoting cancer initiation and progression. Thus, it orchestrates immunosuppression recruiting pro-tumor immune cells of myeloid origin. Furthermore, new recent findings showed that this cytokine can be directly produced by tumor cells in a positive feedback loop and contributes to the failure of targeted therapy. Activation of anti-apoptotic signaling pathways and senescence are some of the mechanisms recently proposed, but the role of IL-1 in tumor cells refractory to standard therapies needs to be further investigated.

## 1. Introduction

Interleukin-1 (IL-1) represents a master cytokine of local and systemic inflammation. It exerts both beneficial, promoting innate immunity against invading microorganisms, and harmful roles in a plethora of autoimmune and autoinflammatory diseases including cancer. A causal relation between inflammation and cancer has been proposed by Virchow in 1863, who hypothesized that malignant neoplasms arise within a region of chronic inflammation causing tissue injuries and increased cell growth [1]. During the last two decades, clinical and epidemiological observations strongly supported Virchow’s hypothesis; it is now clear that inflammation is a key factor involved in all aspects of carcinogenesis mediating initiation, uncontrolled cells proliferation, invasion, angiogenesis and metastasis [2,3,4]. Current estimates suggest that about 25% of epithelial cancers are correlated with chronic inflammation, sustained by infection or inflammatory conditions of diverse origins [5]. Usually, massive inflammation promotes tumor progression and, concomitantly, induces immunosuppression recruiting myeloid cells such as neutrophils, tumor-associated macrophages (TAMs), namely the activated macrophage-polarized M2 phenotype, myeloid-derived suppressor cells (MDSCs), regulatory dendritic cells and regulatory T cells (Treg) [6]; meanwhile, tumor cells undergo immune escape and proliferate rapidly [7]. Because of this interplay, IL-1 has become an attractive therapeutic target and nowadays several inhibitors are available and applied for a wide range of malignancies. One important and emerging debate is the source of IL-1 production and the concept that tumor-derived IL-1 is able to trigger and sustain cancer development. In this review, we will discuss the contribution of the IL-1 receptor family and its ligands in the development of solid tumors and its role in the establishment of drug resistance.

## 2. IL-1 Receptor Family and Its Ligands

IL-1 receptors (IL-1Rs) belong to the Ig-like receptor superfamily characterized by the presence of Toll/interleukin-1 receptor (TIR) domain, which is essential for IL-1 activities. In 1996, the link between TIR domain and innate immunity was identified for the first time. These receptors are now called Toll-like receptors (TLRs) and are known to be involved in the innate immune response [8]. During the last ten years, the IL-1R family has been expanded to co-receptors, decoy receptors, binding proteins, and inhibitor receptors. Particularly, IL-1 family ligands include seven molecules with pro-inflammatory activity: IL-1α, IL-1β, IL-18, IL-33, IL-36α, β and γ. These seven agonists bind three different receptors belonging to the IL-1R family; IL-1α and IL-1β bind IL-1RI (IL-1R1), IL-18 binds IL-18Ra (IL-1R5), IL-33 binds ST2 (IL-1R4), and IL-36α, β and γ bind IL-1Rp2 (IL-1R6). Apart from IL-18 that uses an accessory protein, all the other IL-1 family ligands, through their binding, induce the cognate receptor to form a heterodimer with IL-1RAcP (IL-1R3). Once the complex is made (like IL-1R1/IL-1RAcP/IL-1), the recruitment of the signaling adaptor, myeloid differentiation primary response 88 (MyD88), to the TIR domain initiates the signal cascade by phosphorylation of several kinases. This activation leads to the expression of a large number of inflammatory genes [9,10].

## 3. IL-1: Active Precursors and Dual Function

As regulator of immunological and inflammatory responses, IL-1 exerts a crucial role in mediating autoinflammatory, autoimmune, infectious and degenerative diseases. In the central nervous system, IL-1 induces fever and the activation of the hypothalamus–pituitary–adrenal (HPA) axis. Like the other ligands, IL-1α and IL-1β are encoded by distinct genes. Although they bind the same receptor and show similar biological properties, the impact on inflammation and cancer differs [11]. IL-1α (like IL-33) is active both in its precursor and cleaved forms and it is usually found as a cell-associated cytokine or secreted in the extracellular milieu, exerting a dual-function. The intracellular precursor (pro-IL1α) is constitutively expressed in epithelial layers of the gastrointestinal tract, lung, liver, kidney, endothelial cells, monocyte and astrocytes. It contains a nuclear localization sequence (NLS), responsible for a nuclear localization, where it modulates gene transcription [7,8,9,10]. Upon apoptosis, cytosolic pro-IL-1α translocates into the nucleus and remains tightly bound to the chromatin, failing to induce inflammation. In contrast, in the presence of necrotic signals, cytosolic pro-IL-1α, is released and fully active; it functions as an alarmin by rapidly initiating a cascade of cytokines and chemokines, which account for sterile inflammation [12]. Furthermore, the pro-IL-1α might behave as an oncoprotein since its expression induces neoplastic changes in cells. For example, it has been demonstrated that upon IL-1R1 signaling blockade, pro-IL-1a stimulates IL-8 production in different cells and promotes inflammation [13].

Unlike IL-1α, the IL-1β precursor (pro-IL-1β) is not functionally active. It needs to be cleaved by intracellular caspase-1 or extracellular neutrophilic proteases in order to be active in the extracellular space [10]. Moreover, IL-1β is not expressed in homeostatic conditions, but it is induced upon inflammation and its secretion is tightly controlled at transcription, translation and post translational levels [11]. IL-1β is mainly produced in response to TLR stimuli by hematopoietic cells such as blood monocytes, tissue macrophages, skin dendritic cells and brain microglia [10].

## 4. Role of IL-1 in Solid Tumors

Inflammation is a crucial feature of the malignant phenotype and chronic inflammation is strongly associated with approximately one fifth of all human cancers [1]. IL-1 has been shown to be up-regulated in several types of tumors including breast, colon, head and neck, lung, pancreas and melanomas. In addition, patients with high levels of IL-1 have generally bad prognosis [14,15]. IL-1 can be directly produced by cancer cells or it can “educate” cells, within the tumor microenvironment, to do so [16]. A study conducted by Elaraj et al. proved that melanoma, non-small cells carcinoma, colon, and squamous cancer cell lines exhibit a significant copy number increase in both IL-1α and IL-1β, exerting a paracrine and autocrine action [17]. In line with these findings, a positive association between IL-1 production and metastatic melanoma suggested that endogenous IL-1 acts as a growth factor, enhancing synthesis of other cytokines or chemokines, such as IL-6, TGF-β, IL-8 and adhesion molecules [18,19]. Stromal and myeloma plasma cells, through autocrine stimulation of low doses of IL-1β, trigger IL-6 production, which in turn is responsible for the expansion and survival of myeloma cells [20]. Consistently, fibrosarcoma cell lines, established from tumors derived by Interleukin 1 Receptor Antagonist (IL-1Ra)-deficient mice, were more aggressive and metastatic than tumor cell lines derived from wild-type mice [21]. Thus, the source of inflammation is the tumor itself, since all cancer cells of epithelial origin contain IL-1α in its precursor form [11]. Notably, upon necrotic death consequent to tumors outgrow, IL-1α precursor is readily released and triggers local production of chemokines, which facilitate the recruitment of neutrophils and monocytes [22]. RT-PCR performed in tumor specimens from patients with metastatic colon adenocarcinoma, non-small-cell lung cancer or melanoma revealed high IL-1 gene expression in >50% of all samples tested [17]. The switch-on state of IL-1 genes in malignant cells is likely led by genetic alterations and by microenvironment-derived cues. Indeed, high IL-1 concentrations, within the tumor microenvironment, have been reported in several studies both in cancer patients and experimental models and associated with a more aggressive phenotype [23]. In some tumors, IL-1 and pro-inflammatory cytokines can be up-regulated by oncogenes establishing a favorable environment for the invasiveness of tumor cells. In other types of tumors, instead, IL-1 is stimulated only during the late stage of cancer progression and metastasis formation [24].

In contrast to the above findings, different lines of evidence showed a protective role of IL-1. For example, tumor regression has been observed in vivo in the presence of IL-1 in different types of tumors including sarcoma, melanoma and adenocarcinoma [25,26]. In this context, the anti-tumor effect of IL-1 is related to the ability to induce T helper 1 (Th1) and 17 (Th17) response [27] with an anti-cancer activity. In addition, IL-1 signaling is able to induce anti-tumor effects when activated in specific cell types. Indeed, IL-1R1 deletion in myeloid cells resulted in increased inflammation and enhanced bacterial infiltration, thus boosting CRC tumor progression [28]. Furthermore, in breast cancer, it has been demonstrated that IL-1 maintains metastatic cells in a differentiated state via ZEB1 activation, and inhibition of IL-1R results in metastatic dissemination [29]. For all these controversial results, the development of anti-IL1 cancer treatments needs to be carefully considered in the specific tissue context and mechanistic studies need to be intensively pursued.

### 4.1. IL-1 Is an Inducer of Carcinogenesis

Chronic inflammation is widely recognized as one of the hallmarks of carcinogenesis, tumor progression, and metastasis [30]. The main experimental evidence comes from the mouse model of IL-1β ablation that displayed reduced tumor growth compared to the wild-type, while in knock-out mice for the antagonist IL-1Ra, the tumor developed rapidly and with a sparse leukocyte infiltrate at the site of carcinogen injection [21]. IL-1α is also involved in the suppression of keratinocyte differentiation leading to neoplastic transformation in a cell-autonomous manner [31]. An important experimental proof arrived with the model of 3-methylcholanthrene (3-MCA)-induced carcinogenesis. 3-MCA-induced fibrosarcoma cell lines, from IL-1a-deficient mice, showed that host-derived IL-1α is involved in cancer immunoediting by affecting innate and adaptive immunosurveillance mechanisms [32]. The mechanism by which IL-1 mediates tumorigenesis is not completely understood. One model predicts that immune, epithelial and pre-malignant cells produce DNA damaging molecules such as ROS and NO primed by IL-1 [30,31], linked to the ability of inflammatory cytokines to increase the activity of activation-induced cytidine deaminase (AID) enzyme that causes genomic instability and mutations in many types of cancers [33]. IL-1 from the microenvironment might further contribute to the accumulation of mutations, induced by ROS or NO release, thus rescuing tumor cells from apoptosis and exacerbating the malignant phenotype [34]. Consequently, the effect of IL-1 on carcinogenesis depends on how IL-1, directly or indirectly, orchestrates the cytokine network. The so called “net cytokine effect” is dependent on the local repertoire of cytokines and their receptors in malignant and tumor microenvironment cells and it fluctuates at various phases of tumor development [35].

### 4.2. IL-1 Mediates Angiogenesis and Metastasis

During the initial development, the tumor is dormant until it undergoes the angiogenic switch. IL-1β and the vascular endothelial growth factors (VEGFs) are the main factors responsible in establishing and maintaining tumor-mediated angiogenesis. One of the major mechanisms of the angiogenic switch is an enhanced expression and secretion of angiogenic factors, mainly VEGF, by the malignant cells [23,24]. IL-1 has complex effects on the activation of endothelial cells (ECs) in a prothrombotic/proinflammatory direction by inducing pro-coagulant activity and expression of adhesion molecules and inflammatory cytokines [34]. Several studies provide evidence that angiogenesis and VEGFs are IL-1 dependent [3]. Moreover, IL-1 plays a role in regulating the physiology of ECs, including their activation, increased migration and proliferation and lastly organization into tube-like structures. Finally, IL-1 induces profound changes in gene expression and function allowing these cells to actively participate in inflammatory reactions, immunity, and blood vessel formation [24]. For example, IL-1β stimulates morphological transformation in human dermal micro-vascular endothelial cells accompanied by an increased growth rate, loss of contact inhibition, and increased permeability [36,37]. In addition, IL-1β interacts with IL-1R1 in ECs, inducing cell migration and tube-like structure formation, mainly via activation of p38-mitogen-activated protein kinase (MAPK) and MAPK-activated protein kinase 2 [38]. In vivo, IL-1 plays a synergistic, pro-angiogenic role with VEGF, upregulating growth factor and inflammatory cytokine genes signature in ECs. VEGF/IL-1 stimuli activate genes preferentially through nuclear factor of activated T-cells (NFAT) and NF-kB signaling, respectively [39]. It was also observed that both VEGF and IL-1β increase the permeability of ECs via the Src-dependent pathway [40]. Notably, IL-1β is also essential for endothelial precursor cells (EPCs) to mature into ECs by synergistic interaction with VEGF [24]. The role of IL-1α is less potent as a pro-angiogenic mediator compared to IL-1β, nevertheless, in vivo studies demonstrate that both agonistic molecules are relevant [24].

IL-1α can stimulate a high angiogenic response by recruiting macrophages that are an abundant source of fibroblast growth factor (FGF) or other VEGF-expressing inflammatory cells [41,42]. By in vitro and in vivo studies, IL-1α was shown to stimulate ECs to produce IL-8 and, during in vivo angiogenesis, the source of IL-1 is represented by peripheral blood mononuclear cells (PBMCs) or activated platelets [43]. IL-1α is also released from ECs following stress signals such as starvation or TNF activation. In addition, IL-1α induces ECs to express CXCL1, VCAM-1 and ICAM-1, thus promoting trans-endothelial-migration of inflammatory cells [24]. Activation of angiogenesis and tumor dissemination are closely linked. Indeed, IL-1-treated animals showed increased tumor size along with a strong vascularization, responsible for the invasion of the underlying muscle tissue. These tumors also showed a strong positive reaction with intercellular adhesion molecule-1 antibody [44]. On the contrary, a reduction in the volume of subcutaneous B16 murine melanoma tumors was observed in mice treated with IL-1Ra, which also showed decreased size and number of liver and lung metastasis and improved survival [45]. In line with this, IL-1α/β KO mice failed to develop solid tumor following injection of melanoma cells and exhibited improved survival compared to wild type animals, which died due to lung metastasis [23]. Moreover, a significant correlation between IL-1α expression and distant metastasis was found in patients with head and neck squamous cell carcinoma [46]. Cell motility is a fundamental and ancient cellular behavior that contributes to metastasis. In this regard, the role of IL-1 has been elucidated. For example, in melanoma cell line, IL-1 significantly improves migration by IL-1RI/II pathways [47]. In addition, NF-kB, activated by IL-1, increases the migratory activity of breast cancer cells and upregulates CXCL8 under oxygen deprivation [48]. Finally, in a breast cancer mouse model it was demonstrated that IL-1 signaling is responsible for tumor progression and metastasis and this effect was ameliorated by blocking IL-1R signaling [49].

### 4.3. IL-1 Is Responsible for Immunosuppression

In the tumor microenvironment (TME), IL-1, produced by tumor cells, stromal elements or infiltrating leukocytes, is involved in the modulation of anti-tumor immunity. Specifically, in the dominant immunosuppression TME, MDSCs, TAM, tumor associated neutrophils (TAN), regulatory B (Breg) cells and Th17 were reported as sources of IL-1 and can be regulated by IL-1 itself [6,15]. It has been proven that tumor-derived soluble factors and IL-1β are important stimuli for the expansion and migration of MDSCs [50]. These cells downregulate immune surveillance and antitumor immunity by multiple mechanisms. The importance of IL-1β as a driver of MDSC propagation is underlined by the finding that, in peripheral blood of advanced melanoma patients, an increase in IL-1β in the serum was associated with heightened frequency of monocytic MDSCs and Treg [51]. Furthermore, IL-1β upregulates COX-2, which encodes prostaglandins that mediate MDSCs propagation. MDSCs produce IL-1β and other pro-inflammatory molecules driving tissue-resident endothelial cells to produce VEGF and other angiogenic factors [52]. Macrophages, particularly, populate the tumor microenvironment and are highly prevalent in inflammation-mediated tumors. These cells, named activated (M2) macrophages, infiltrate tumor tissues and are the major source of inflammasome activation and IL-1β production [53]. In this context, increased IL-1β provides a nurturing niche for cancer stem cells, promoting angiogenesis and metastasis formation, and taming adaptive immune response [34]. A key role of IL-1, in the differentiation of Th17 cells from naïve T cells and the subsequent maintenance of their phenotype, has been proven both in vitro and in vivo [54]. For example, IL-1 enables robust Th17 development, thus the latter can be inhibited by IL-1 blockade [55]. In line with this, Il1r1^−/−^ mice displayed a decreased Th17 cell response following immunization [56]. A strong immune infiltration was recently reported in a subset of colorectal cancer patients, belonging to the Consensus Molecular Subtypes 1 (CMS1), a subgroup of patients characterized also by high mutation rate and microsatellite instability (MSI) [57]. As a confirmation, gene expression profiling of CMS1 showed evidence of a strong immune activation (immune response, PD1 activation, NK cells, Th1 cell and cytotoxic T cell infiltration signatures), consistent with pathological descriptions of prominent tumor-infiltrating CD8+ cytotoxic T lymphocytes [58]. This subclass of tumors is characterized by high expression of genes specific to Treg cells, MDSCs, monocyte-derived cells and Th17 cells. Moreover, CMS1 tumors display a marked upregulation of immunosuppressive factors, such as TGF-β and CXCL12, and high expression of genes encoding chemokines that attract myeloid cells, including C-C motif chemokine ligand 2 (CCL2) and the related cytokines IL-23 and IL-17, which are known carcinogenic drivers in colitis-associated CRC [59].

The concept of targeting immunosuppression fostered the development of checkpoint blockade immunotherapy which, so far, has shown some exciting clinical successes in specific cancer types. The therapeutic potential of checkpoint blockade is clearly remarkable; nevertheless, only a subset of patients benefit from these agents and, generally, they are not designed to target the oncogenic elements of tumor inflammation, like cytokines with pro-growth and/or pro-survival functions. Therefore, although the manipulation of cytokines pathways may be clinical in its own right, combining this approach with checkpoint blockade may yield even greater advantages by simultaneously unleashing antitumor immunity and blocking the pro-tumorigenic elements of inflammation [60]. These reasons involve fostering the rational for a combinatorial blockade of PD-1/PD-L1 and IL-1, due to the expected synergistic effect on tumor growth. In this regard, Kaplanov et al. proved that a combined neutralization of IL-1β and PD-1 was responsible for an astonishing abrogation of tumor development in a mammary carcinoma murine model [61]. Similar results were obtained in a model of ductal pancreatic adenocarcinoma, in which IL-1β interception significantly enhances the antitumor activity of PD-1 blockade [62].

## 5. IL-1 and Resistance to Targeted Therapy

Recently, a critical role for the IL-1 pathway, in relation to therapy, is emerging in different types of solid tumors [63,64,65,66]. Specifically, several studies proposed a role for IL-1 in the poor responses to EGFR blockade and radiotherapy [67] with consequent treatment failures. An increased production of IL-1 was detected in head and neck squamous cell carcinoma (HNSCC), less responsive to an anti-EGFR kinase inhibitor (erlotinib). In addition, the driver role of IL-1 was proven and the treatment with IL-1 antagonist anakinra was sufficient to overcome this phenotype [68]. Subsequently, the same authors demonstrated that suppression of Myd88 expression blocked erlotinib-induced IL-1 secretion in vitro and in vivo [69]. In non-small cell lung cancer (NSCLC), IL-1β-induced EH domain-containing protein 1 (EHD1), potentiating EGFR-TKI resistance and epithelial-to-mesenchymal transition (EMT) [70]. In line with this, treatment with BRAF inhibitors (BRAFi) such as dabrafenib and vemurafenib, used in melanoma patients with BRAFV600E mutation, strongly upregulated IL-1β production in myeloid mouse antigen presenting cells (APC). The suggested mechanism depends on dabrafenib activation of the inflammasome that induces caspase-8 activation and pro-IL-1β processing. An alternative mechanism explaining BRAFi treatment-induced tolerance, in melanoma, is represented by a cytokine-signaling network involving TAM-derived IL-1β and CAFs-derived CXCR2 ligands [15]. IL-1 was reported to be secreted by tumors of colon-cancer xenopatients, who poorly responded to the therapy with anti-EGFR monoclonal antibody. Specifically, IL-1α/β abundance, along with their cognate receptor IL-1R1 expression, predicts both response to therapy and disease relapse-free survival and it appears intriguingly associated to the CMS1 [71,72].

In cancer cells, certain therapeutic agents can induce cell-cycle arrest in the form of senescence, the so called therapy-induced senescence (TIS) [73]. In this setting, a pro-inflammatory secretome is established, namely senescence-associated secretory phenotype (SASP), where IL-1 has been proven to be an upstream regulator [74]. Long-term exposure to SASP initiates EMT [75] and stemness, escaping from TIS in a mechanism dependent on the WNT/β -catenin pathway and returning to the cell cycle, while maintaining stemness properties [76]. The ability of cancer cells to acquire a stem-like phenotype is a hallmark in tumors. Studies of molecular mechanisms have shown that IL-1β promotes the stemness of HNSCC and melanoma cells, through the activation of Smad/ID1 signalling pathways and the upregulation of stemness factor genes (Bmi1 and Nestin), thus increasing drug resistance [29,77]. This phenotype is critically associated with tumor progression and therapy failure.

For all the reasons described so far, in the clinical practice intercepting IL-1β/IL-1α or the cognate receptor is becoming a valuable therapeutic approach. For instance, MABp1, a true human moAb anti-IL-1α, showed disease control in patients with 18 different tumor types and its efficacy was further confirmed, in 2017, in a randomized, double-blind, placebo-controlled phase 3 study in a cohort of 333 advanced colorectal cancer patients [78]. In line with this, intercepting IL-1β with canakinumab significantly reduced the incidence of lung cancer in a cohort of 10,061 patients [79]. An independent cohort of 47 patients with smoldering/indolent myeloma, treated for six months with anakinra and low dose of dexamethasone, displayed progression-free disease that lasted over 3 years and, and in 8 patients, even over 4 years [80]. These studies provide a good rationale for an early use of anti-cytokine therapy in combination with kinase inhibitors or anti-immunosuppressive agents in cancer treatment. As a consequence, a number of clinical trials testing the efficacy of anti-IL1 therapy in cancer are currently recruiting patients. A list of the most relevant trials was reported in Table 1. To sum up, the IL-1 pathway is seen as a potent inducer of inflammation by activating and sustaining a feed-back loop of pro-inflammatory cytokine release, which may promote drug resistance and tumor survival.

## 6. Conclusions

Defense against endogenous and exogenous danger signals is a mechanism shared among all living organisms in the form of innate immunity, while less than 5% depend on adaptative immunity. Inflammation is a hallmark of innate immunity and, if uncontrolled, can be detrimental for survival [81,82,83]. During the last decades, the role of chronic inflammation in solid tumors has become evident not only as a risk factor but also as a trigger for a favorable tumor environment [84]. IL-1, in this context, has emerged as a culprit involved in all aspects of tumor development including carcinogenesis, angiogenesis and metastasis formation. Notably, the role of IL-1 is not only limited to tumor growth promotion, but it is also emerging as a prognostic factor for patients in response to targeted therapy. This is confirmed by the finding that, in different types of tumors, patients with high levels of IL-1 commonly have a bad prognosis. Furthermore, treatment with targeted therapy, TKIs and/or moAbs, has been shown to fail due to IL-1 production. Thus, the use of anti-cytokine treatments in combination with kinase inhibitors and immunosuppressive blockade may produce beneficial effects, as proven in recent in vivo studies in breast, pancreas and lung cancer. However, the mechanism through which IL-1 is involved in a poor response to targeted therapy remains to be completely elucidated.

**Figure 1:**
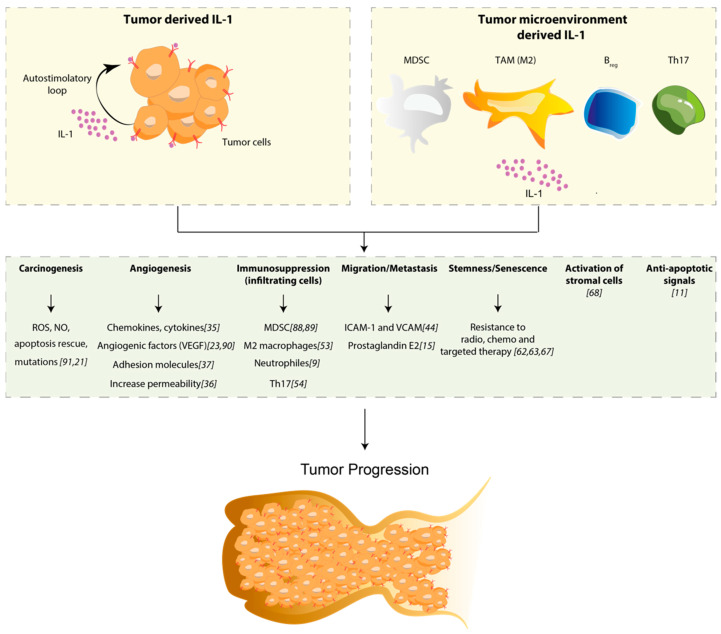
Schematic representation of the role of IL-1 in tumor progression. The upper panel represents two sources of IL-1. Specifically, the tumor-derived IL-1 (upper left) establishes an auto-stimulatory loop that sustains IL-1 synthesis and release, while the tumor microenvironment-derived IL-1 (upper right) arises from immune cells of the myeloid origin: myeloid-derived suppressor cells (MDSC), macrophage-polarized M2 phenotype, regulatory B (Breg) and T helper (Th17) cells. As shown in the figure, the boost of IL-1, released from both tumor and immune cells, induces a wide range of effects: it sustains the production of Reactive Oxygen Species (ROS), and Nitric Oxide (NO) that exacerbate mutation rate rescuing cells from apoptosis; it increases the production of chemokines, cytokines and all adhesion molecules responsible for vascular permeability, leading to angiogenesis and metastasis. It recruits immunosuppressive cells (MDSC, M2 macrophages, neutrophils and Th17) and activates stromal cells in the TME [88,89,90,91]. Finally, IL-1 is described to be involved in drug resistance through the induction of anti-apoptotic signals and senescence.

During the last decades, the role of IL-1 in the TME has been widely investigated [85,86,87]. IL-1 exerts its function modulating the composition of TME by recruiting MDSCs, TAM, TAN, Breg cells and Th17, and these subpopulations of immune cells are the source of IL-1 themselves. This particular TME, along with the increased IL-1 production, has a harmful role, establishing a positive feedback loop that augments local inflammation and exerts a positive impact in tumor growth and drug resistance. Several mechanisms through which IL-1 influences tumor progression and drug resistance have been described. In particular, tumor microenvironment-derived-IL-1 promotes: (a) proliferation and survival by sustaining the production of ROS, NO and mutation occurrence; (b) metastasis dissemination by fostering the expression of adhesion molecules like I-CAM and V-CAM; (c) angiogenetic switch by increasing the production of cytokine, chemokines and facilitating endothelial permeability; (d) the activation of anti-apoptotic signals, which rescue apoptotic cells from death. Nevertheless, recent findings suggest that, in addition to the TME, the tumor itself is a direct source of IL-1, contributing to tumor growth and drug resistance. This notion has a logical explanation since all cancer cells of epithelial origin contain IL-1 in its precursor form that, consequently to tumors outgrowth, will be readily available, upon necrotic death, or processed in its active form through inflammasome activation. Tumor-derived IL-1 has been proven to have a role in recruiting and expanding MDSC, contributing to the angiogenic switch sustaining the production of angiogenic factors such as VEGF (Figure 1). As mentioned, IL-1 is also emerging as a cytokine involved in drug resistance; we recently reported that increased levels of tumor-derived IL-1 are correlated with cetuximab resistance in colorectal cancer xenopatients and that abundance of IL-1R1 is predictive of therapy response [72]. These latest results lay the groundwork of a new perspective for IL-1 and, on the basis of the recent pre-clinical and clinical data, it is already clear that combining anti IL-1 agents with checkpoint inhibitors could represent a promising strategy against solid tumors.

## Figures and Tables

**Table 1:** Ongoing clinical trials testing anti-IL-1 drugs, either alone or in combination, in cancer therapy.

Therapy	Target	Tumor Type	Recruitment Status	Development status	ClinicalTrial.gov Identifier	Sponsor	Start Date	Estimated Completition Date
Anakinra + Everolimus	IL-1 Ra + anti mTOR	Neoplasm	Active, not recruiting	Phase 1	NCT01624766	M.D. Anderson Cancer Center	June, 2012	June, 2020
Anakinra + Chemo	IL-1 Ra + anti mTOR	Pancreatic Adenocarcinoma	Active, not recruiting	Early Phase 1	NCT02550327	Baylor Research Institute	January, 2016	August, 2023
Anakinra + JCARH125	IL-1 Ra + CAR T-cells	Multiple Myeloma	Recruiting	Phase 2	NCT03430011	Juno Therapeutics	March, 2023	March, 2023
Anakinra	IL-1 Ra	Multiple Myeloma	Active, not recruiting	Phase 2	NCT03233776	Radboud University	May, 2019	June, 2020
Anakinra	IL-1 Ra	Multiple Myeloma	Recruiting	Phase 2	NCT04099901	Radboud University	October, 2020	October, 2022
Anakinra + Axicabtagene Ciloleucel	IL-1 Ra + CAR T-cells	Neoplasm, Large B-Cell Lymphoma	Not yet recruiting	Phase 1,2	NCT04432506	M.D. Anderson Cancer Center	July, 2020	January, 2025
Anakinra + Axicabtagene Ciloleucel	IL-1 Ra + CAR T-cells	B-Cell Non-Hodgkin Lymphoma	Not yet recruiting	Phase 2	NCT04359784	Fred Hutchinson Cancer Research Center	August, 2020	December, 2021
Anakinra + Axicabtagene Ciloleucel	IL-1 Ra + CAR T-cells	Non-Hodgkin Lymphoma	Not yet recruiting	Phase 2	NCT04150913	Marcela V. Maus, M.D.;Ph.D.	July, 2020	November, 2024
Anakinra	IL-1 Ra	B-Cell Lymphoma and Non-Hodgkin Lymphoma	Recruiting	Phase 2	NCT04148430	Memorial Sloan Kettering Cancer Center	October, 2019	October, 2022
Anakinra + Axicabtagene Ciloleucel	IL-1 Ra + CAR T-cells	Large B-Cell Lymphoma	Recruiting	Phase 2	NCT04205838	Jonsson Comprehensive Cancer Center	March, 2020	December, 2022
Canakinumab	mAb anti IL-1β	Non-small Cell Lung Cancer	Recruiting	Phase 3	NCT03447769	Novartis Pharmaceuticals	March, 2018	January, 2027
Canakinumab + Spartalizumab + LAG525	mAb anti IL-1β + mAb anti PD-1+ mAb anti LAG-3	Triple Negative Breast Cancer	Recruiting	Phase 1	NCT03742349	Novartis Pharmaceuticals	January, 2019	January, 2022
Anakinra +/− Pembrolizumab	mAb anti IL-1β +/− mAb anti PDL-1	Non-small Cell Lung Cancer	Recruiting	Phase 2	NCT03968419	Novartis Pharmaceuticals	November, 2019	January, 2022
Anakinra + Pembrolizumab + Chemo	mAb anti IL-1β +/− mAb anti PDL-1	Non-small Cell Lung Cancer	Active, not recruiting	Phase 3	NCT03631199	Novartis Pharmaceuticals	December, 2018	September, 2022
Canakinumab + PDR001	mAb anti IL-1β + mAb anti PD-1	Triple Negative Breast Cancer and NSCLC	Active, not recruiting	Phase 1	NCT02900664	Novartis Pharmaceuticals	August, 2016	August, 2020
Canakinumab + Spartalizumab	mAb anti IL-1β + mAb anti PD-1	Renal Cell Carcinoma	Recruiting	Early Phase 1	NCT04028245	Charles G. Drake	August, 2019	December, 2021
Canakinumab	mAb anti IL-1β	Myelodysplastic Syndrome or Chronic Myelomonocytic Leukemia	Not yet recruiting	Phase 2	NCT04239157	M.D. Anderson Cancer Center	June, 2020	December, 2021
Canakinumab + PDR001 + Chemo	mAb anti IL-1β + mAb anti PD-1	Non-small Cell Lung Cancer	Active, not recruiting	Phase 1	NCT03064854	Novartis Pharmaceuticals	May, 2017	December, 2021
Canakinumab + Spartalizumab	mAb anti IL-1β + mAb anti PD-1	Melanoma	Recruiting	Phase 2	NCT03484923	Novartis Pharmaceuticals	September, 2018	June, 2022
Canakinumab + Chemo	mAb anti IL-1β	Non-small Cell Lung Cancer	Active, not recruiting	Phase 3	NCT03626545	Novartis Pharmaceuticals	January, 2019	March, 2022
Xilonix + Chemo	mAb anti IL-1α	Pancreatic cancer	Active, not recruiting	Phase 1	NCT03207724	Andrew Hendifar, MD	October, 2017	December, 2020
CAN04 + Pembrolizumab	mAb anti IL1RAP+mAb anti PD-1	Non-Small-Cell Lung, Urothelial CarcinomaMalignant Melanoma, Head and Neck Squamous Cell Carcinoma	Not yet recruiting	Phase 1	NCT04452214	Cantargia AB	September, 2020	January, 2022
CAN04 + Chemo	mAb anti IL1RAP	Non Small Cell Lung Cancer, Pancreatic Ductal Adenocarcinoma, Triple Negative Breast Cancer, Colorectal Cancer	Recruiting	Phase 1/2	NCT03267316	Cantargia AB	September, 2017	June, 2021

## Abbreviations

IL-1	Interleukin-1
TAM	Tumor-associated macrophage
MDSC	Myeloid-derived suppressor cell
IL-1R	Interleukin-1 receptor
Th17	T helper 17
Treg	Regulatory T cell
Breg	Regulatory B cell
TLR	Toll like receptor
MyD88	Myeloid differentiation primary response 88
VEGF	Vascular endothelial growth factor
TAN	Tumor-associated neutrophil
EC	Endothelial cell
